# Preoperative concurrent chemotherapy with S-1 and radiotherapy for locally advanced squamous cell carcinoma of the oral cavity: Phase I trial

**DOI:** 10.1186/1756-9966-29-33

**Published:** 2010-04-20

**Authors:** Hiroyuki Harada, Ken Omura

**Affiliations:** 1 Department of Oral Surgery, Oral Restitution, Division of Oral Health Sciences, Graduate School, Tokyo Medical and Dental University, Japan; 2 Department of Advanced Molecular Diagnosis and Maxillofacial Surgery, Hard Tissue Genome Research Center, Tokyo Medical and Dental University, Japan

## Abstract

**Background:**

This study was conducted to identify a recommended dose for S-1, used in combination with 40-Gy radiation.

**Methods:**

Thirty patients, 15 each with stage III and IVA oral carcinoma, were enrolled. All patients received a total dose of 40-Gy. For the S-1 treatment, patients were given either the standard Japanese dose, calculated according to body surface area, or a reduced dose. Groups consisting of at least three patients were given S-1 according to one of 8 regimens.

**Results:**

Hematologic toxicity was mild and reversible. The most common nonhematologic toxicity was mucositis. At level 8 that was the standard S-1 dose for 5 days per week for 4 weeks, dose-limiting toxicity was observed when 2 patients had grade 4 mucositis. This level was thus deemed the maximum tolerated dose for the regimen.

**Conclusions:**

The recommended dose of S-1 with concurrent radiotherapy was the reduced dose of S-1 given for 5 days per week, for 4 consecutive weeks. Preoperative S-1 and concurrent radiotherapy was well tolerate and feasible and warrants a phase II study.

## Introduction

Today the treatment of primary oral squamous cell carcinoma includes various combinations of radiotherapy, chemotherapy and surgery. In literature searches, studies employing adjuvant strategies of radiotherapy after surgery outnumber those of preoperative concepts. Nevertheless, for about 20 years, preoperative therapy concepts have been established as the standard approach in some centers. Klug et al. summarized the results of the preoperative chemoradiotherapy for oral cancer [1]. He reported that 5-year survival rate determined by the meta-analysis of the 32 studies (1927 patients) was 62.6%, appearing to be remarkably good.

Kirita et al. reported obtaining a clinical response rate of 97.9%, and a 5-year overall actuarial survival rate of 81.3%, by treating advanced oral cancer with preoperative concurrent cisplatin- or carboplatin-based intravenous chemotherapy and radiotherapy at a total dose of 40-Gy [2]. Iguchi et al. reported an overall response rate of 100% when treating oral and maxillary carcinoma with concurrent chemoradiotherapy, using a combination of intraarterial pirarubicin, intravenous continuous 5-fluorouracil (5-FU), and a radiation dose of 40-Gy [3]. They concluded that their concurrent chemotherapy regimen is effective as a preoperative modality, with a remarkably high response rate and an acceptable level of adverse events.

S-1 is an oral fluoropyrimidine preparation that consists of tegafur, 5-chloro-2, 4-dihydroxypyridine (gimeracil), a dihydropyrimidine dehydrogenase (DPD) inhibitor, and potassium oxonate (oteracil), which inhibits orotate phosphoribosyl transferase in the gastrointestinal tract, thereby reducing the gastrointestinal toxicity of 5-FU [4]. A preclinical study showed that gimeracil, a DPD inhibitor, is a potent radiosensitizing agent [5]. Preclinical studies using human oral cancer xenograft models showed a better response from a combination of S-1 and fractionated radiotherapy than from either treatment alone [6].

Here, we report a phase I study of S-1 chemotherapy performed concomitantly with a radiation dose of 40-Gy as the preoperative treatment for oral squamous cell carcinoma. The purpose of this study was to identify the maximum tolerated dose (MTD) of S-1 in combination with 40-Gy radiation, the dose-limiting toxicity (DLT) of S-1, and the recommended dose (RD) for this treatment.

## Patients and Methods

### Patient eligibility

Previously untreated patients with histopathologically proven oral squamous cell carcinoma of stage III or IVA were evaluated for this study. Eligible patients were required to be from 20 to 75 years old, have an Eastern Cooperative Oncology Group performance status of 0 or 1, life expectancy of at least 3 months, and adequate organ function (leukocytes ≧ 4000/mm^3^, platelets ≧ 100,000/mm^3^, hemoglobin level ≧ 9.0 g/dl, aspartate aminotransferase (AST) level ≦ 2 times the upper normal limit (UNL), alanine aminotransferase (ALT) level ≦ 2 times the UNL, alkaline phosphatase (ALP) level ≦ 2 times the UNL, serum bilirubin ≦ 1.5 mg/dl, and serum creatinin ≦ the UNL.

Patients were excluded if they had received any prior systemic chemotherapy or radiotherapy, had a concomitant malignancy, active inflammatory bowel disease, active gastric/duodenal ulcer, active infection, severe heart disease, mental disorder, or other severe concurrent disease. Pregnant or lactating females were also excluded.

The protocol was approved by the Institutional Review Board of Tokyo Medical and Dental University. All patients gave written informed consent before entry into this study.

### Treatment

**Figure 1:**
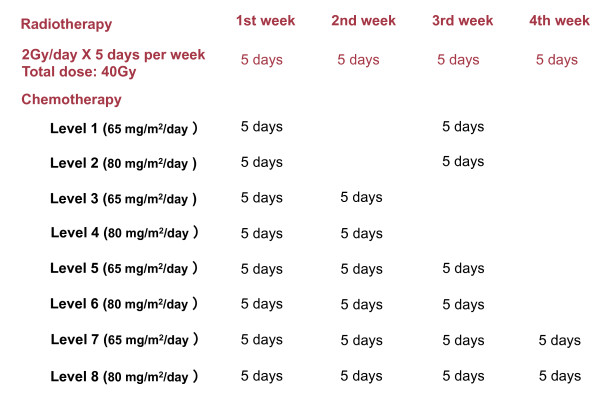
**Administration schedule**.

We gave a fractional daily dose of 2-Gy for 5 days a week to a total dose of 40-Gy using a 4-MV LINAC to deliver X-rays to the primary tumor site, and if the patients had nodal disease, to the cervical nodes (Figure 1).

S-1 (Taiho Pharmaceutical Co., Tokyo, Japan) was administered orally twice a day after meals, concomitant with radiotherapy. Each capsule of S-1 contained either 20 or 25 mg of tegafur, and individual doses, calculated according to body surface area (BSA), were rounded down to the nearest pill size. The dosing of S-1 was as follows (standard dose, reduced dose): BSA < 1.25 m^2^, 80 mg or 50 mg daily; BSA ≧ 1.25 m^2 ^but < 1.5 m^2^, 100 mg or 80 mg daily; BSA ≧ 1.5 m^2^, 120 mg or 100 mg daily. S-1 was administered to patients on 5 consecutive days per week, following the schedules shown (Figure 1).

Adverse events were evaluated according to the National Cancer Institute Common Toxicity Criteria, version 2.0. Hematological DLT was defined as grade 4 leukopenia or neutropenia, grade 3 febrile neutropenia, or grade 3 thrombocytopenia. Nonhematologic DLT was defined as grade 4 mucositis, or grade 3 or 4 nonhematological toxicities (excluding nausea/vomiting). The dosing plan was, if none of 3 patients in a treatment group experienced DLT at a given dose level, the dose of S-1 was escalated in the next cohort of 3 patients. If one of the initial 3 patients experienced DLT, 3 patients were added at the same dose level. Dose escalation continued if DLT was observed in only one of 6 patients. If 2 or more patients experienced DLT at the dose level, the dose of that level would be the MTD.

Our initial protocol consisted of dosing schedules to level 6 (Figure 1) in September 2002, but no DLT was observed even at level 6. Therefore, we added levels 7 and 8 in January 2005. Meanwhile, three patients were enrolled in level 6. Consequently level 6 consisted of six patients. In determining the RD, we considered the practical aspects of administering S-1 in addition to the manifestations of toxicity.

### Treatment evaluation

All patients underwent surgery after chemoradiotherapy, but the follow-up periods were not adequate for treatment effects. Therefore, we judged the clinical efficacy of the chemoradiotherapeutic protocol immediately just before surgery. The median interval between the end of chemoradiotherapy and surgery was 26.0 days (range, 15-48 days).

The evaluation methods included computed tomography (CT) scan, magnetic resonance imaging (MRI), and ultrasound. Responses at the primary site and the neck were analyzed separately. Treatment effects were estimated based on changes in tumor size. A complete response (CR) was defined as the complete clinical and radiologic disappearance of the primary tumor. The neck response was deemed complete with the disappearance of any adenopathy, as determined using CT and ultrasound. A partial response (PR) was characterized as a 50% or greater decrease in the product of two perpendicular diameters of the primary and regional tumors by the time of surgery. Stable disease (SD) was defined as a tumor reduction of less than 50%. Progressive disease (PD) was indicated by an increase of 25% or more in the volume of any tumor or the appearance of new lesions.

For the histological evaluation of primary tumors, we used Shimosato's classification of therapeutic effectiveness [7]. Grade 0 indicates no noticeable change; grade I, minimal cellular changes present, but the majority of tumor cells appear viable; grade IIa, despite the presence of cellular changes and partial destruction of the tumors, the tumor is still readily recognizable, and a many tumor cells appear viable; grade IIb, the tumor destruction is extensive, but viable cell nests are present in small areas of the tumor (one-quarter of the tumor mass, excluding areas of coagulation necrosis); grade III, only a few scattered, markedly altered, presumably nonviable tumor cells are present, singly or in small clusters, and few or no viable cells are seen; grade IV, no tumor cells remain in any section.

### Statistical Analysis

Survival time was assessed from the first day of treatment until death or the last patient contact. Overall survival and cumulative survival rates were calculated according to the Kaplan-Meier method [8].

## Results

### Patient characteristics

Thirty patients, 24 men and 6 women, were enrolled in this study between September 2002 and January 2007. Their median age was 58.5 years (range, 32-75 years) and their ECOG score was 0 for 29 patients and 1 for a patient. The primary lesion sites were the tongue (n = 10), the floor of the mouth (n = 4), the upper gum (n = 5), the lower gum (n = 9), and the buccal mucosa (n = 2). The TN classification is shown in Table 1. Fifteen patients each had stage III or IVA carcinomas. The median follow-up period was 67 months (range 37-89 months).

**Table 1:** TN classification

	T2	T3	T4a	Total
N0	0	7	2	9
N1	5	3	2	10
N2b	2	4	3	9
N2c	0	0	2	2

Total	7	14	9	30

### Toxicity

Cases with toxicities observed during treatment or within 2 weeks after chemoradiotherapy are listed in Additional file 1. Grade 1-2 leukocytopenia was observed in 46.7% (n = 14) of the patients. Neutropenia was rare; grade 1-2 neutropenia occurred in 5 patients (16.7%). Grade 1 anemia was observed in 60% (n = 18) of the patients and grade 1 elevated AST in 40% (n = 12). For all treatment levels, the hematologic toxicity was grade 1 or 2. Generally, the hematologic toxicity was mild and reversible, and there was no grade 3 or 4 hematologic toxicity.

Nonhematological toxicities, apart from mucositis, were grade 1 or 2, and the most common was mucositis. Grade 1 or 2 mucositis was observed at treatment levels 1-4. Although 11 patients (36.7%) had grade 3 mucositis, there was no DLT at levels 1-7. One of three patients experienced a DLT (grade 4 mucositis) at level 8: based on the results, three additional patients were added, one DLT was seen. Consequently, 2 DLTs were observed among 6 patients at level 8, thus the doses used level 8 were deemed the MTD in this study. Therefore, we propose the level 7, the reduced S-1 dose 5 days per week for 4 weeks, as the RD.

### Efficacy

The clinical responses of the primary tumors are shown in Table 2. Three patients achieved CR and 25 achieved PR. The overall clinical response rate (CR or PR) was 93.3%. The histological evaluation was grade IV (no viable tumor cells in any section) in 2 patients (Table 3) and grade III in 13. The histological response rate, defined as grades of IIb, III, or IV, was 90.0%.

**Table 2:** Clinical response of the primary tumors

	CR	PR	SD	PD	Response rate
Level 1		3			100%
Level 2	1	2			100%
Level 3	1	2			100%
Level 4		3			100%
Level 5		3			100%
Level 6		4	2		66.7%
Level 7		3			100%
Level 8	1	5			100%

Total	3	25	2	0	93.3%

Abbreviations: CR = complete response, PR = partial response, SD = stable disease, PD = progressive disease

**Table 3:** Histologic evaluation of the primary tumors after chemoradiotherapy

	IV	III	IIb	IIa	I	Response rate
Level 1		2	1			100%
Level 2	1	2				100%
Level 3		2	1			100%
Level 4	1	2				100%
Level 5		1	2			100%
Level 6			4	1	1	66.7%
Level 7		1	1	1		66.7%
Level 8		3	3			100%

Total	2	13	12	2	1	90.0%

*The histological evaluation was defined according to Shimosato's classification of therapeutic effectiveness

Clinical responses of neck metastases are shown in Table 4. Nine patients showed clinical PR, 10 showed SD, and 2 showed PD. The clinical response rate (CR or PR) of the neck disease was 42.9%.

**Table 4:** Clinical response of the neck disease

	CR	PR	SD	PD	Response rate
Level 1		1	1		50%
Level 2			1	1	0%
Level 3		1	2		33.3%
Level 4		2	1		66.7%
Level 5			3		0%
Level 6		1	1	1	33.3%
Level 7		3			100%
Level 8		1	1		50%

Total		9	10	2	42.9%

Abbreviations: CR = complete response, PR = partial response, SD = stable disease, PD = progressive disease

After surgery, local failure developed in one patient (level 6), and neck failure and distant metastasis occurred in another (level 7). With a median follow-up of 67 months, the 5-year overall survival rate was 90.0%, and the 5-year cumulative survival was 93.1%.

## Discussion

We set out to determine the safety and reliability of concurrent S-1 and radiotherapy in advanced cancer of the oral cavity, in a phase I study. Many studies have demonstrated that combined chemotherapy and radiation is a highly effective treatment modality for increasing the survival of patients with advanced disease [2,3,9-11]. Concurrent chemoradiotherapy has been established as an appropriate standard for many patients with locally advanced head and neck cancer.

To the best of our knowledge, this study is the first trial of S-1 and radiotherapy in oral cancer. Tsukuda et al. reported that most adverse events of S-1 administration alone were hematological, gastrointestinal, and skin toxicities, although most of these were grade 1 or 2 and controllable [12]. In the present study, there was no severe hematological, gastrointestinal, or skin toxicity. Mucositis was the most common adverse event, with grade 3 mucositis observed in 66.7% of patients at levels 5, 6, and 7 (Additional file 1). Grade 4 mucositis, constituting DLT, was observed in 2 of 6 patients at level 8. The doses used level 8 was deemed the MTD. Therefore, the determined recommended dose of S-1 was the reduced dose for 5 days per week for 4 weeks (level 7).

In a multi-institutional cooperative late phase II clinical study of S-1 alone in patients with advanced/recurrent head and neck cancer in Japan, the clinical response rate of the primary tumor was 36.4% in oral cancer patients [13]. In the present study, the overall clinical response rate was 93.3%, and the histological response rate was 90.0%, appearing to be remarkably good.

Many studies have demonstrated concurrent chemoradiotherapy to be effective in patients with advanced head and neck cancer. However, the majority of studies have reported total radiation doses of more than 60-Gy. Tsukuda et al. reported that the complete response rate were 93% in stage III and 54% in stage IV, by treating head and neck cancer with S-1 and radiotherapy at a total dose of 66-70.2 Gy [14]. There have been few reports on the effect of preoperative chemoradiotherapy with a total radiation dose of 40-Gy [2,3].

Osteoradionecrosis of the jaw is one of the serious complications of radiotherapy for head and neck cancer. High-dose radiotherapy for oral cancer induces mandibular osteoradionecrosis with an incidence of approximately 5% to 20% [15,16]. The management of osteoradionecrosis is difficult and not always successful. Therefore, if the antitumor effect could be increased by combining chemotherapy with lower doses of radiotherapy, it might reduce radiation-related adverse events without sacrificing efficacy. The combined method studied here has the potential to increase the antitumor effect while minimizing surgery. Therefore, a phase II study is warranted.

On the other hand, the clinical response rate for neck nodal disease was 42.9%. This result was poor compared with the clinical response rate of the primary tumor. A late phase II clinical study of S-1 alone found a clinical response rate was 21.7% for cervical lymph node metastasis [13]. These results have suggested that neck dissection is warranted for metastatic lymph nodes in patients with oral carcinoma.

In conclusion, the concurrent administration of S-1 and radiotherapy was well tolerated and yielded sufficiently positive results. The RD of S-1 with concurrent radiotherapy for this protocol is BSA <1.25 m^2^, 50 mg/day; BSA 1.25-1.5 m^2^, 80 mg/day; BSA ≥ 1.5 m^2^, 100 mg/day for 5 days per week for 4 weeks. We have already started a phase II study in multiple institutes.

## Conflict of interests

The authors declare that they have no competing interests.

## Authors' contributions

HH carried out clinical data collection, data review, participated in study design. KO was the principle investigation of the study and participated in all aspects of this work. All authors read and approved the final manuscript.

## Supplementary Material

Additional file 1 Prevalence of adverse events Click here for file
