# Supplementary material for: Preoperative concurrent chemotherapy with S-1 and radiotherapy for locally advanced squamous cell carcinoma of the oral cavity: Phase I trial
Source: J Exp Clin Cancer Res. 2010 Apr 20;29(1):33. doi: 10.1186/1756-9966-29-33 (PMC2867809; doi:10.1186/1756-9966-29-33)
Supplement: Additional file 1 — Prevalence of adverse events [file 1756-9966-29-33-S1.DOCX]

Additional file 　　　　　　　　　　　　　　　　　　　　　　Prevalence of adverse events

| Toxicity* | Level 1 (n=3) | | | | Level 2 (n=3) | | | | Level 3 (n=3) | | | | Level 4 (n=3) | | | | Level 5 (n=3) | | | | | Level 6 (n=6) | | | | | | Level 7 (n=3) | | | | | Level 8 (n=6) | | | |
| --- | --- | --- | --- | --- | --- | --- | --- | --- | --- | --- | --- | --- | --- | --- | --- | --- | --- | --- | --- | --- | --- | --- | --- | --- | --- | --- | --- | --- | --- | --- | --- | --- | --- | --- | --- | --- |
|  | G1 | G2 | G3 | G4 | G1 | G2 | G3 | G4 | G1 | G2 | G3 | G4 | G1 | G2 | G3 | G4 | G1 | G2 | G3 | G4 | G1 | | G2 | | G3 | | G4 | G1 | | G2 | G3 | G4 | G1 | G2 | G3 | G4 |
| Hematological toxicity | | | | |  | | | |  | | | |  | | | |  | | | | |  | | | | | |  | | | | |  | | | |
| Leukocytopenia | 2 | 0 | 0 | 0 | 0 | 0 | 0 | 0 | 0 | 1 | 0 | 0 | 1 | 0 | 0 | 0 | 1 | 0 | 0 | 0 | 2 | | | 1 | | 0 | 0 | 3 | 0 | | 0 | 0 | 1 | 2 | 0 | 0 |
| Neutropenia | 0 | 0 | 0 | 0 | 0 | 0 | 0 | 0 | 1 | 0 | 0 | 0 | 0 | 0 | 0 | 0 | 0 | 0 | 0 | 0 | 0 | | | 0 | | 0 | 0 | 2 | 0 | | 0 | 0 | 0 | 2 | 0 | 0 |
| Hemoglobin | 2 | 0 | 0 | 0 | 1 | 0 | 0 | 0 | 3 | 0 | 0 | 0 | 2 | 0 | 0 | 0 | 2 | 0 | 0 | 0 | 4 | | | 1 | | 0 | 0 | 2 | 0 | | 0 | 0 | 2 | 0 | 0 | 0 |
| Thrombocytopenia | 1 | 0 | 0 | 0 | 0 | 0 | 0 | 0 | 0 | 0 | 0 | 0 | 0 | 0 | 0 | 0 | 0 | 0 | 0 | 0 | 1 | | | 0 | | 0 | 0 | 0 | 0 | | 0 | 0 | 0 | 0 | 0 | 0 |
| AST | 1 | 0 | 0 | 0 | 1 | 0 | 0 | 0 | 0 | 0 | 0 | 0 | 0 | 0 | 0 | 0 | 2 | 0 | 0 | 0 | 2 | | | 0 | | 0 | 0 | 2 | 0 | | 0 | 0 | 4 | 0 | 0 | 0 |
| ALT | 1 | 0 | 0 | 0 | 0 | 0 | 0 | 0 | 0 | 0 | 0 | 0 | 0 | 0 | 0 | 0 | 2 | 0 | 0 | 0 | 1 | | | 0 | | 0 | 0 | 1 | 0 | | 0 | 0 | 3 | 1 | 0 | 0 |
| Nonhematological toxicity | | | | |  | | | |  | | | |  | | | |  | | | | |  | | | | | |  | | | | |  | | | |
| Anorexia | 1 | 2 | 0 | 0 | 0 | 1 | 0 | 0 | 1 | 0 | 0 | 0 | 0 | 0 | 0 | 0 | 1 | 1 | 0 | 0 | 0 | | | 3 | | 0 | 0 | 0 | 3 | | 0 | 0 | 2 | 4 | 0 | 0 |
| Fatigue | 1 | 0 | 0 | 0 | 1 | 0 | 0 | 0 | 0 | 0 | 0 | 0 | 0 | 0 | 0 | 0 | 1 | 0 | 0 | 0 | 3 | | | 1 | | 0 | 0 | 3 | 0 | | 0 | 0 | 1 | 0 | 0 | 0 |
| Dermatitis | 3 | 0 | 0 | 0 | 2 | 0 | 0 | 0 | 2 | 1 | 0 | 0 | 1 | 2 | 0 | 0 | 0 | 3 | 0 | 0 | 4 | | | 2 | | 0 | 0 | 1 | 2 | | 0 | 0 | 0 | 6 | 0 | 0 |
| Mucositis | 0 | 3 | 0 | 0 | 1 | 2 | 0 | 0 | 1 | 2 | 0 | 0 | 0 | 3 | 0 | 0 | 0 | 1 | 2 | 0 | 0 | | | 2 | | 4 | 0 | 0 | 1 | | 2 | 0 | 0 | 1 | 3 | 2 |

* Toxicities were defined according to the National Cancer Institute Common Toxicity Criteria, version 2.0.

Abbreviations: G=grade, AST=aspartate aminotransferase, ALT=alanine aminotransferase
